# Developing Novel Experimental Models of m-TORopathic Epilepsy and Related Neuropathologies: Translational Insights from Zebrafish

**DOI:** 10.3390/ijms24021530

**Published:** 2023-01-12

**Authors:** Murilo S. de Abreu, Konstantin A. Demin, Maria M. Kotova, Foad Mirzaei, Sanobar Shariff, Burhan Kantawala, Ksenia V. Zakharchenko, Tatiana O. Kolesnikova, Karen Dilbaryan, Artem Grigoryan, Konstantin B. Yenkoyan, Allan V. Kalueff

**Affiliations:** 1 Institute of Chemical Technology, Ural Federal University, 620002 Yekaterinburg, Russia; 2 Institute of Translational Biomedicine, St. Petersburg State University, 199034 St. Petersburg, Russia; 3 Moscow Institute of Physics and Technology, 141701 Moscow, Russia; 4 Neuroscience Laboratory, COBRAIN Center, Yerevan State Medical University Named after Mkhitar Heratsi, Yerevan 0025, Armenia; 5 Neurobiology Program, Sirius University of Science and Technology, 354340 Sochi, Russia; 6 Pathophysiology Department, Yerevan State Medical University Named after Mkhitar Heratsi, Yerevan 0025, Armenia; 7 Laboratory of Preclinical Bioscreening, Granov Russian Research Center of Radiology and Surgical Technologies, Ministry of Healthcare of Russian Federation, 197758 St. Petersburg, Russia; 8 Almazov Medical Research Centre, Institute of Experimental Medicine, Ministry of Healthcare of Russian Federation, 197341 St. Petersburg, Russia

**Keywords:** mTOR, signaling pathway, zebrafish, animal model, mTORopathy

## Abstract

The mammalian target of rapamycin (mTOR) is an important molecular regulator of cell growth and proliferation. Brain mTOR activity plays a crucial role in synaptic plasticity, cell development, migration and proliferation, as well as memory storage, protein synthesis, autophagy, ion channel expression and axonal regeneration. Aberrant mTOR signaling causes a diverse group of neurological disorders, termed ‘mTORopathies’. Typically arising from mutations within the mTOR signaling pathway, these disorders are characterized by cortical malformations and other neuromorphological abnormalities that usually co-occur with severe, often treatment-resistant, epilepsy. Here, we discuss recent advances and current challenges in developing experimental models of mTOR-dependent epilepsy and other related mTORopathies, including using zebrafish models for studying these disorders, as well as outline future directions of research in this field.

## 1. Introduction

**Figure 1:**
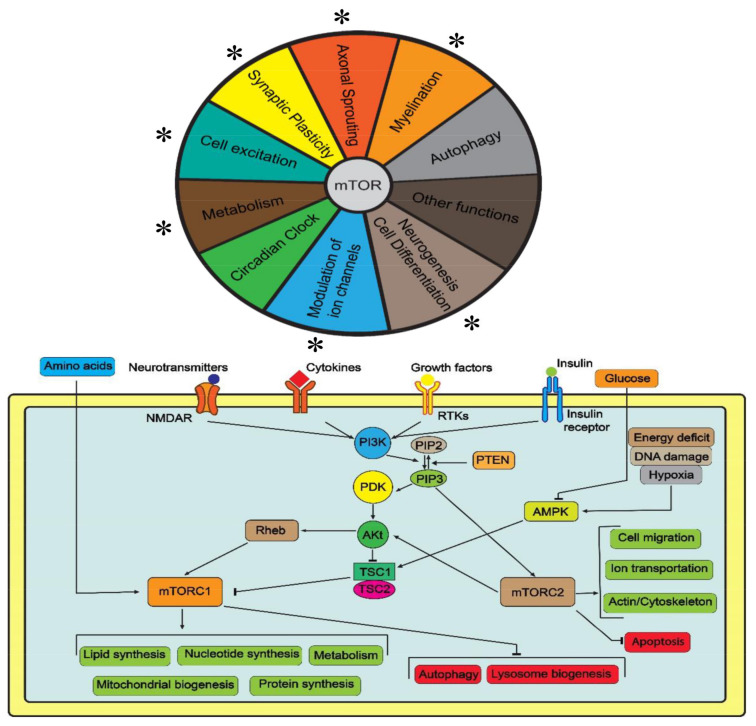
A brief summary of the mTOR signaling pathway, its mediation of cellular processes and their relevance to epilepsy (* denotes high relevance).

The mammalian target of rapamycin (mTOR) is an important molecular sensor of cellular metabolism and a key regulator of cell growth and proliferation [1]. mTOR is a serine/threonine protein kinase forming two protein complexes, mTORC1 and mTORC2 (Figure 1). Acting to promote cell growth, mTORC1 consists of mTOR itself, regulatory-associated protein of mTOR (RAPTOR) and several other cellular proteins. Positively regulated by the presence of amino acids, oxygen and energy supply, and inhibited by rapamycin, mTORC1 reduces autophagy and activates anabolism (e.g., protein, lipid and nucleotide synthesis) for protein synthesis, mainly via phosphorylation of ribosomal protein S6 by protein kinase 1 (S6K1, to increase ribosome biogenesis) and eukaryotic translation initiation factor 4E (eIF4E)-binding protein 1 (4E-BP1, to stimulate mRNA translation) [2,3].

mTORC1 stimulates synthesis of nucleotides required for DNA replication and ribosome biogenesis [4] (Figure 1), as well as the ‘activating transcription factor 4′/ATF4-dependent expression of methylenetetrahydrofolate dehydrogenase NADP+-dependent 2 (MTHFD2), to provide mitochondrial tetrahydrofolate and one-carbon units for purine synthesis. Additionally, S6K1 phosphorylates and activates carbamoyl phosphate synthetase for de novo pyrimidine synthesis [5,6]. Through lipin 1, mTOR modulates sterol regulatory-element binding protein (SREBP), a master regulator of lipo- and steroidogenic gene transcription, thus activating cellular lipogenesis [7]. mTORC1 also promotes the translation of hypoxia-inducible factor 1-alpha (HIF1α), hence stimulating the expression of glucose transporters and glycolysis [8]. In contrast, the tuberous sclerosis complex (TSC) proteins TSC1 (hamartin) and TSC2 (tuberin), encoded by two tumor suppressor genes *TSC1* and *TSC2*, accordingly, form the TSC1-TSC2 complex that negatively regulates mTORC1 [9].

mTORC2 is a large rapamycin-insensitive complex of mTOR with several cellular proteins that regulates cell survival, proliferation and migration by phosphorylating and activating protein kinase B (Akt), the main mediator of insulin signaling, and several protein kinases (e.g., PKA, PKC and PKG) [10]. As this requires anabolic support, mTORC2 interacts with the TSC1-TSC2 complex to activate mTOR and collaboratively promote cell proliferation [11,12] (Figure 1 and Table 1).

Due to critical biological roles of mTOR in vivo, its deficient signaling causes a diverse group of neurological disorders, termed ‘mTORopathies’. Typically arising from mutations within the mTOR signaling pathway, these severe neurological disorders are characterized by overt neuromorphological abnormalities and prominent, often treatment-resistant, epilepsy (Table 1 and Figure 1). Here, we discuss recent advances and current challenges in developing experimental models of mTOR-dependent epilepsy and other related mTORopathies, including using the zebrafish (*Danio rerio*), as well as outline future directions of research in this field.

## 2. The Role of mTOR in the Nervous System

**Figure 2:**
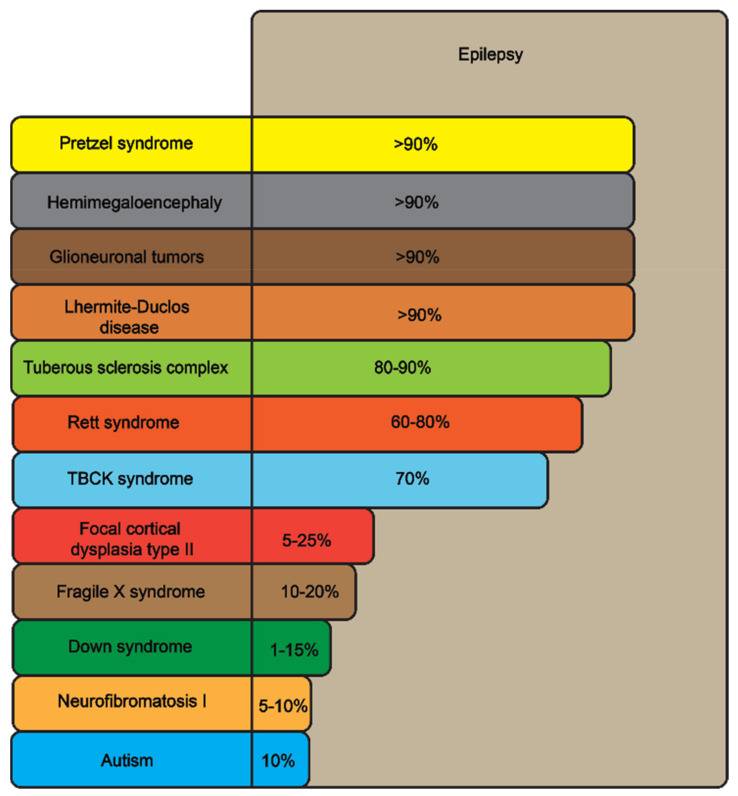
Epilepsy as a key shared clinical feature of multiple disorders associated with deficient mTOR signaling (also see Table 1 for details), based on relative occurrence (estimated as % of affected patients with epilepsy).

mTOR is abundantly expressed in the brain, especially in the hippocampus, striatum, amygdala and prefrontal cortex [13,14]. Brain mTOR plays a crucial role in cellular excitability, synaptic plasticity, cell growth, neuronal migration, development and proliferation, as well as in memory storage, protein synthesis, autophagy, neurogenesis, dendritic growth/arborization, ion channel expression and axonal regeneration [15,16,17]. Aberrant mTOR signaling strongly contributes to the pathogenesis of mTORopathies and is directly linked to epilepsy associated with these disorders (Table 1 and Figure 2).

### 2.1. Autophagy

As an evolutionarily conserved, lysosomal-driven catabolic response to malnutrition, autophagy is inhibited by trophic factors (the TSC-Ras homolog enriched in brain/RHEB pathway) or amino acid supply (the phosphoinositide 3-kinase/PI3K-RHEB pathway) that activate mTOR. In contrast, the mTOR inhibition by low amino acid availability, energy stress (via AMP-activated protein kinase, AMPK) or rapamycin, activates autophagy [18]. Both increased and reduced autophagy can trigger neurotoxicity and cause neurodegenerative disorders [19]. Moreover, autophagy regulates the structural composition of myelin, as specific deletion of *Atg7* (an essential autophagy gene) in Schwann cells results in the accumulation of excess cytoplasm and organelles, together with small fiber hypermyelination [20].

### 2.2. Axonal Sprouting and Synaptic Plasticity

Since ephrin-A ligands activate neuronal ephA receptors, mitogen-activated protein kinase (MAPK) ERK1/2 activity becomes inhibited, hence reducing the TSC2 inhibition [21]. As ephrin stimulation increases TSC2 activity and inactivates the mTOR pathway, ephrin-induced inhibition of neuronal mTORC1 is critical for axonal guidance [21]. In contrast, the extracellular matrix protein reelin activates mTORC1 to control dendritic outgrowth and branching [16]. Calmodulin-dependent protein kinase CaMKII is used by mTOR to improve dendritic arborization in the hippocampus, whereas downstream activation of S6K1 increases dendritic arborization in the prefrontal cortex (PFC) [22]. Hippocampal long-term potentiation (LTP), a physiological index of synaptic plasticity, also requires mTOR, thereby implicating it in memory and cognition via the protein synthesis-dependent stabilization of synapses [18]. In contrast, altered synaptic and structural plasticity impaired serotonergic function, and memory/learning deficits are associated with upregulated neuronal PI3K-Akt/mTOR signaling [17].

### 2.3. Neurogenesis, Cell Differentiation and Myelination

mTORC1 controls the maintenance of neural stem cells (NSCs), neuronal differentiation and migration, as well as axonal and dendritic growth, that contribute to both neurodevelopment and adult neurogenesis [16]. NSCs give rise to progenitors and ultimately neuroblasts in the subventricular (SVZ) and subgranular (SGZ) zones of the hippocampus in adult brain [23]. Constitutive neurogenesis emerges from phosphatase and tensin homolog (*PTEN*) deletion in adult SVZ NSCs, which activates the Akt-mTORC1 pathway. The enhancer of zest homolog2 (Ezh2), a component of polycomb repressive complex 2 that primarily silences genes by methylating H3K27 stimulates the proliferation of NSCs in the SGZ by inhibiting *PTEN* expression and activating the Akt-mTOR signaling [19]. In contrast, inhibition of mTOR signaling may contribute to the degradation of cortical neurons, likely underlying neurological problems seen in children exposed to prenatal inflammation [24].

The role of mTOR signaling in regulating myelination is supported by *Raptor* or *Rheb1* deletion that blocks mTORC1 function, *Rictor* ablation that blocks the mTORC2 function, and *mTOR* ablation that impairs both complexes [25]. Compared to only disrupting mTORC1, hypomyelination and molecular alterations are more robust when mTORC2 activity is also abolished [26,27]. Interestingly, instead of showing altered number of oligodendrocytes, transgenic mice overexpressing Akt produce CNS hypermyelination (i.e., more myelin synthesized per oligodendrocyte), which requires the Akt/mTOR signaling to increase the translation of myelin mRNAs [28].

### 2.4. Regulation of Ion Channels and Other Functions

Local Kv1.1 production is inhibited by glutamate N-methyl-D-aspartate (NMDA) receptor activation through mTORC1, most likely by NMDA-mediated PI3K activation after Ca^2+^ entry. This positive feedback mechanism may promote the activation of voltage-gated Na^+^ and/or K^+^ channels, helping generate the action potentials [19].

The circadian clock is a fundamental physiological mechanism that synchronizes behavioral and physiological activity, controlled in mammals by the hypothalamic suprachiasmatic nucleus (SCN) [19]. 4E-BPs and S6Ks (S6K1 and S6K2) are regulated by mTORC1 activated by light. The detachment of 4E-BP from eIF4E and the activation of cap-dependent translation result from 4E-BP phosphorylation. Likewise, the translation of vasoactive intestinal peptide (VIP) mRNA is controlled by 4E-BPs, whereas another regulatory mechanism involves photic ERK/MAPK activation, which phosphorylates eIF4E via MNK kinases and encourages Per1 and Per2 mRNA translation in the SCN. Thus, to control mRNA translation in the SCN, the MAPK and mTOR pathways must interact with eIF4E [29], providing a general regulation of circadian clocks. 

Hypothalamic mTOR in the arcuate nucleus is also implicated in the regulation of food intake to control body energy balance, since centrally administered leucine reduces appetite and body weight while boosting hypothalamic mTOR signaling [18]. Finally, the initiation of puberty and the gonadotropic axis are both regulated by centrally expressed mTOR [30]. 

## 3. Clinical mTORopathies

As already noted, human mTORopathies represent a diverse group of neurological conditions, most prominently involving epilepsy and cortical maldevelopment [31,32] (Table 1 and Figure 2). Pathological changes to myelin are also associated with aberrant mTOR signaling, along with reduced oligodendroglia content and the loss of precursor cells [33]. For example, inadequate myelination of cortical neurons due to disrupted mTOR signaling is a key pathogenic factor in drug-resistant seizures, whereas decreased myelin composition is associated with both the relative length of epilepsy and a higher mTOR expression [34]. Aberrant mTOR signaling is also strongly linked to neurodevelopmental and neurodegenerative diseases [34]. For instance, TSC, neurofibromatosis type 1 (NF1) and fragile X syndrome are all caused by mutations of mTOR-inhibiting genes (Table 1) [35], whereas rapamycin-induced mTOR inhibition prevents the proliferation of cytomegalic neurons and reduces episodic convulsions [36].

Tuberous sclerosis, or TSC, is a systemic neurological disorder caused by mutations in *TSC1* or *TSC2* that lead to benign tumors in the brain and other tissues. Clinically, TSC is a rare autosomal dominant condition that presents as seizures, behavioral/developmental abnormalities, neuroectodermal nodules and subependymal giant cell astrocytomas [37]. Presently the most well-studied mTORopathy (Table 1), TSC involves poor myelination and fewer oligodendrocytes in and around cortical lesions in affected patients [38] (also see similar phenotype in oligodendrocyte-specific loss of *TSC1* gene in rodent models of TSC [39]).

A spontaneous anatomical abnormality of the neocortex, focal cortical dysplasia type II (FCDII) is characterized by treatment-resistant epilepsy due to atypical neurons and dyslamination caused by deficient mTOR signaling [40]. Another severe mTORopathy, beta-propeller protein-associated neurodegeneration (BPAN), is associated with iron accumulation and neurodegeneration, presenting as intellectual disability, psychomotor retardation, febrile seizures, autism, progressive cognitive loss, dementia, dystonia and parkinsonism [41]. Polyhydramnios, megalencephaly and symptomatic epilepsy (PMSE), or Pretzel syndrome, is a rare brain disorder characterized by macrocephaly, craniofacial dysmorphism, infantile-onset treatment-resistant epilepsy and cognitive decline. This disorder is caused by a single mutated gene encoding STE20-related kinase adaptor alpha (STRADA), a potent upstream inhibitor of mTOR via AMPK [31] (Table 1).

Hemimegaloencephaly (HEM) causes enlarged, asymmetrical heads in children, who also develop epilepsy, paralysis and delayed cognitive development [42]. HEM occurs due to de novo somatic mutations in genes encoding key components of the mTOR signaling pathway, PI3K catalytic subunit (PI3KCA), Akt3 and mTOR [43]. Glioneuronal tumors (GNTs) are low-grade neuroepithelial tumors that typically affect children and young adults, presenting as early-onset focal epilepsy. In BRAF V600E mutant GNTs, LKB1-induced phosphorylation of LKB1 may impact the mTOR activity, potentially by dissociating the LKB1-AMPK-mTOR signaling [44]. The mTOR pathway expression correlates with the severity of gliomas, and almost all glioblastomas have mutations that affect PI3K or PTEN [45].

Lhermitte–Duclos disease (LDD) is an extremely rare, hereditary slow-growing cerebellar gangliocytoma due to aberrant mTOR signaling caused by mutated mTOR-inhibiting phosphatase and tensin homolog deleted on chromosome ten (*PTEN)* gene. LDD involves thickened folia and replacement of the internal granular layer by abnormal ganglion cells in the cerebellum and presents as hamartoma tumor syndrome, ataxia and seizures [46]. The TBCK syndrome is another very rare neurogenetic disorder that involves mutant TBC1 domain containing kinase (TBCK) gene (an activator of the mTOR signaling) and presents clinically as leukoencephalopathy, coarse face, neuropathy, epilepsy, hypotonia and intellectual disability [47]. 

Overall, mTORopathies markedly increase individual susceptibility to seizures, collectively implicating the mTOR system in epileptogenesis [32]. Another important pathogenetic aspect of mTORopathies is the aberrant upregulation of Tau protein. The initial period of its expression during neurodevelopment corresponds to aberrant neuronal growth and morphogenesis where the mTOR pathway is inactive. Infantile tauopathies have been identified as cortical embryonic developmental tumor gangliogliomas, as well as FCDII, HME and TSC, already discussed here, and are all strongly epileptogenic [48]. 

## 4. Recurrent Behavioral Abnormalities, mTOR and Epilepsy

As already noted, mTOR potently regulates the development of axons and dendrites, as well as the production of proteins that affect neuronal plasticity and memory (Figure 1). Autism spectrum disorder (ASD) is a highly prevalent, hereditary neurodevelopmental disorder that manifests as deficits in communication (both verbal and nonverbal), cognitive decline and repetitive behaviors (behavioral perseverations) [49]. Albeit a highly polygenic brain disorder and not a ‘true’ mTORopathy per se, ASD has neuronal deficiencies linked to mTOR dysregulation [50,51], and epilepsy is frequently comorbid with ASD, further supporting the putative shared pathogenetic link between mTOR deficits, epilepsy and neurodevelopmental disorders in general.

A key pathogenetic factor of inherited mental retardation is the fragile X syndrome (FXS), caused by the mutated fragile X mental retardation gene (*FMR1*) that encodes the fragile mental retardation protein (FMRP), an inhibitor for PI3K enhancer (PIKE, an upstream stimulator of PI3K within the mTOR pathway) [52]. A clinically relevant-to-mTORopathy condition, FXS often manifests as epilepsy [53]. Likewise, the primary cause of Rett syndrome is aberrant methyl-CpG binding protein 2 (MECP2) gene [54], whose production is regulated by the mTOR pathway but (unlike other mTORopathies discussed here) is linked to reduced mTOR signaling. Finally, Down syndrome, the most prevalent inherited developmental condition with mental impairment, often manifests as behavioral perseverations and epilepsy [55]. In the brain of Down patients, the core components of the mTOR pathway are generally hyperactivated, as initial hippocampal expansion amplifies the Akt-mTOR cascade and elevates the expression of phosphorylated proteins S6, 4E-BP1, S6K and mTOR [52]. Aberrant activation of Akt-mTOR signaling in the frontal cortex and hippocampus of Down patients also suggests imbalances in autophagy regulation and mitochondrial dysfunctions [56].

## 5. The Key Role of mTOR in Epilepsy

Because multiple mTORopathies are commonly associated with epilepsy (Figure 2), mTOR signaling emerges as an important factor in its pathogenesis [18,57,58,59,60] in several different ways. First, as already mentioned, mTORopathies often involve malformations of cortical development (Table 1), which may trigger epilepsy by disrupting the normal balance between various excitatory and inhibitory cell types, thereby shifting the overall balance between cortical excitatory and inhibitory neurotransmission. At the same time, the available medications often emerge as being just symptomatic treatments, reducing neuronal excitability but not affecting disorder pathogenesis per se [61]. Thus, searching for novel ways of epilepsy treatment, including drugs that would specifically target mTORopathic deficits, becomes critical. 

For example, rapamycin and other mTOR inhibitors exhibit overt antiepileptic action, whereas hyperactivated mTOR signaling typically triggers epileptogenesis [62]. However, mTOR inhibitors seemingly do not directly inhibit neuronal excitability by binding to ion channels or neurotransmitter receptors, but rather exert their effects indirectly, e.g., through altering the expression of K^+^ channels, glutamate receptors [63,64], neuroplasticity and synaptic morphology [65]. Thus, the exact mechanisms of how mTOR inhibitors contribute to epilepsy, as well as molecular cascades involved, remain yet unclear. 

Indeed, genetic mutations in *TSC1* or *TSC2*, *PTEN* or *STRADA* that encode upstream regulators of mTOR, increase mTOR activity [37,66], whereas the inhibition of mTOR signaling lowers experimental seizures [67,68,69]. However, the role of the downstream mTOR pathway in epilepsy pathogenesis is far less studied. For example, mTORC1 phosphorylates CLIP-170 (CAP-GLY domain containing linker protein 1) that elevates CLIP-170 microtubule-binding properties [70], whose downregulation recovers microtubule arrangement in *Tsc2*^−/−^ cells [71]. Because microtubular organization plays an important role in both neurons and its synapses, its disruption by CLIP-170 is in line with the link between aberrant mTOR pathway and epilepsy. Likewise, chronic administration of rapamycin reduces cortical excitability [72], further supporting this potential pathogenetic link. Interestingly, only chronic, but not acute, rapamycin reduces overall neuronal excitability, including increased gamma aminobutyric acid (GABA)-ergic synaptic activity, rheobase and hyperpolarized membrane potentials [72]. Furthermore, mTOR inhibitors may also exert their effects through the prevention or reversal of abnormal cell growth and increased autophagy in some conditions, given the link of TSC1 and TSC2 or PTEN loss of function to neuronal hyperexcitation [73].

mTORC1 also phosphorylates the signal transducer and activator of transcription 3 (STAT3) [74], an important mediator of cytokines signaling, including interleukins (IL) IL-6 and IL-10, that further acts as a transcription factor altering gene expression [75]. Interestingly, STAT3 is also activated in reactive astrocytes in rat hippocampus injected with kainic acid (a chemoconvulsant) [76], whereas acquired epilepsy can also be caused by viral CNS infections, including viral encephalitis and human herpesvirus 6 [77,78,79,80]. Taken together, this highlights the importance neuroinflammatory-related pathways in mTOR-related signaling in general, potentially implicating it in epilepsy pathogenesis as well.

## 6. Animal Models of mTORopahic Epilepsy

### 6.1. Selected Rodent Models

Experimental (animal) models, especially based on rodents, are a valuable tool in translational neuroscience research [81], helping to target a wide range of brain disorders, including mTORopathies and epilepsy [82] (see Table 2 for details). For example, young adult male B6-129S4-Tsc2tm1Djk/J mice represent a useful transgenic model of TSC with *Tsc2* haploinsufficiency and region-specific alterations in the expression of multiple synaptic proteins [83]. These mice display increased presynaptic VAMP1/2 and phospho-synapsin-1 (Ser62/67) immunoreactivity, along with serious ultrastructural anomalies (e.g., a fuzzy synaptic density structure, more synaptic vesicles, delaminated myelin and larger, heavier brain), elongated swollen synaptic mitochondria (markers of their degradation), distended Golgi apparatus and polyribosomes in the cytoplasm (indicative of aberrant protein metabolism) and altered behaviors (e.g., novel object recognition deficits) [83]. 

Likewise, as Pretzel syndrome is linked to *Strada* mutation, neuronal deficits are seen in CRISPR-edited *Strada* mouse N2a cells, a germline *Strada* knockout mouse, and induced pluripotent stem cell (iPSC)-derived neurons from patients with Pretzel syndrome bearing *Strada* mutation [31]. The iPSC-derived neurons from Pretzel syndrome individuals show increased cell size and hyperactive mTOR signaling, subtle changes in neuronal firing, a more depolarized resting membrane potential and a lower threshold for action potential generation following *Strada* knockout in vitro. Within the subcortical white matter of *Strada* knockout mouse brains, the observed ectopic neurons resemble those seen in human Pretzel syndrome brain tissue [31]. 

Overall, rodent-based animal models (Table 2) are widely used to probe various central aspects of mTOR-related epilepsy and other mTORopathies, especially since the mTOR pathway represents a logical candidate for preclinical screening of antiepileptic medications, given its control of a wide range of cellular processes (from protein synthesis to cell growth, proliferation and synaptic plasticity) involved in epileptogenesis (Figure 1), and because mTOR inhibitors typically slow the onset of epilepsy and prevent other brain abnormalities underlying epilepsy in rodent models of mTORopathies [84].

### 6.2. General Overview of Zebrafish Models of Epilepsy

Complementing rodent studies, the zebrafish has emerged as a powerful vertebrate model organism to assess the role of CNS genes and their associated molecular networks in normal and pathological brain functioning [86,87]. Zebrafish have high genetic and physiological homology to humans, efficient ways for genetic manipulation, inexpensive maintenance, comparable neuroanatomy, and a fully sequenced genome [88]. Zebrafish have transparent embryos and larvae that grow externally and quickly, enabling direct observation of brain activity and neurodevelopmental mechanisms in a fully functional nervous system in vivo. Both adult and larval zebrafish are also highly traceable, making it possible to conduct highly efficient pharmacological screening on a larger scale compared to rodent models [89]. While zebrafish are particularly suitable for large-scale phenotypic analyses (due to their small size and ease of manipulation), their complex, well-defined and context-specific behavior can be reliably assessed in multiple sensitive and well-established behavioral tests [88,90]. Currently, both larval and adult zebrafish are widely used to probe brain malfunctions and their genetic and pharmacological control, as well as in high-throughput CNS drug screening [88]. 

Zebrafish are also commonly used for modeling various epilepsy-related conditions [91]. For instance, experimental epilepsy can be evoked in zebrafish genetically (e.g., spontaneous seizures in genetically modified mutant or knockdown fish) [92] or pharmacologically, by treating with chemoconvulsants, such as picrotoxin, pentylenetetrazole, caffeine or strychnine (e.g., drug-induced seizures) [93]. Complementing a conventional ‘gold standard’ method in epilepsy research—electroencephalography (EEG) — to register epileptic spikes in both larval and adult zebrafish [94], useful physiological biomarkers of epilepsy-related neuronal activation include assessing brain expression of early proto-oncogenes (e.g., *c-fos, c-jun* and *egr1*) in zebrafish brain [95]. 

Other important tools for evaluating zebrafish epilepsy involve phenotypic behavioral screening in both adult and larval fish [96,97,98]. For example, during drug-induced epilepsy, a typical zebrafish seizure-like phenotype includes hyperlocomotion, twitching, abnormal body position, ataxia, high-velocity erratic movements as well as circular and corkscrew swimming [97,98]. Behavioral hyperactivity-related endpoints (typically increased during seizures) include total distance traveled, velocity, turn angle and angular velocity, easily recorded by video-tracking software and particularly valuable for high-throughput antiepileptic drug screening [96]. Other atypical behaviors, such as spasms, unstable bode posture (ataxia) and overt clonic and/or tonic seizures, if recorded, can also represent useful signs of zebrafish epilepsy [98,99]. 

Furthermore, the development of information technology and its application to zebrafish behavioral phenotypes [100,101] may offer new opportunities for unbiased characterization of seizures in both larvae and adult fish. For example, simultaneous recording of fish behavior with two cameras enables reconstructing patterns of fish locomotion in 3D [102] and, if combined with machine learning protocols (e.g., [101]), may promote automatic recognition of various types of epilepsy-related behaviors in fish by artificial neural networks. 

Another highly promising method for studying functional activity of zebrafish brain during epilepsy is light sheet microscopic imaging, based on layer-by-layer CNS photography to assess region-specific activity of larval fish [103]. For example, Ca^2+^ imaging can be used to visualize and record real-time epileptiform activity in zebrafish brain and identify neural circuits involved in epilepsy [104]. The combination of imaging techniques with genomic studies and automated behavioral analysis may also be a promising multivariate approach to evaluate the complex phenotypes of zebrafish epilepsy.

### 6.3. Zebrafish Models of mTORopathic Epilepsy

Mounting evidence suggests zebrafish as a useful model organism to study mTORopathies. For example, the mTOR-inhibiting drug rapamycin markedly reduces pentylenetetrazole-induced tonic–clonic seizures in larval, juvenile and adult zebrafish [105]. Corroborating an important role of PI3K in PI3K/Akt/mTOR signaling and epilepsy further, a selective inhibitor of PI3K, LY294002, exerts a similar anticonvulsant effect in zebrafish treated with pentylenetetrazole, reducing both motor (clonic seizures) and molecular (*c-fos* brain expression) biomarkers of epilepsy in larval fish [106]. 

In addition to pharmacological models, genetic models have also been developed for mTORopathic epilepsy in zebrafish. For instance, using the CRISPR-Cas9 system to generate single-gene mutant zebrafish strains, the Epilepsy Zebrafish Project has extensively evaluated electrophysiological, behavioral, neuroanatomical, survival and pharmacological phenotypes of larval fish mutants for the gene encoding an mTOR-inhibiting protein, *Strada* [107]. These zebrafish display unprovoked electrographic seizure activity (epilepsy), thus supporting their potential as a genetic model of mTORopathic epilepsy (e.g., Pretzel syndrome associated with human *Strada* mutations) and a screen for novel antiepileptic therapies.

Generated to model TSC, genetically modified zebrafish with a nonsense mutation (vu242) in *tsc2* produce a truncated tuberin unable to inhibit Rheb and the TOR kinase within TORC1 [108]. These fish display increased brain cell size, elevated TORC1 signaling and poorly organized forebrain neurons that resemble brain malformations in human TSC [108]. Homozygous *tsc2*^−/−^ mutant zebrafish larvae display enlarged brains, reduced locomotor behavior and epileptiform discharges, markedly reduced by pericardially injected rapamycin [109]. Likewise, *tsc2*-deficient zebrafish recapitulate anatomical and behavioral signs of clinical TSC, including aberrant brain morphology, thinning brain connections, epileptogenesis and anxiety-like behavior, which are rescued by reducing TrkB signaling [110]. Furthermore, cannabidiol treatment reduces the number and size of phosphorylated rpS6-positive cells in larval *tsc2*^−/−^ zebrafish brain, suggesting opioid-mediated suppression of mTOR activity in this genetic model [111], which may also be relevant to antiepileptic effects of opioids in drug-resistant seizures seen clinically [112,113].

Reduced mTOR inactivation is a key trigger of epilepsy in both clinical and animal studies. DEP domain-containing protein 5 (DEPDC5) is a component of the amino acid-sensing Gap Activity TOward Rags GATOR1 complex, acting as a negative regulator of mTOR signaling. DEPDC5 mutations often result in various focal epilepsies, and DEPDC5 knockdown zebrafish show spontaneous epileptiform activity, increased susceptibility to drug-induced seizures, general hypoactivity, premature death and overall hyperactivation of mTOR signaling [114]. Epilepsy-like hyperkinesia, circular swimming and increased neuronal activity in these fish persist throughout embryonic development and are significantly reduced upon treatment with rapamycin [114]. Interestingly, immunostaining of DEPDC5 mutant zebrafish brain for GABAergic markers shows specific defects in the fine branching of the GABAergic network, which are rescued by rapamycin [115]. These findings not only reinforce the role of mTOR in zebrafish models of epilepsy, but also link it to aberrant GABA signaling. In line with this, mTORC1 hyperactivity, early lethality and ventricular dilatation in the DEPDC5^−/−^*tsc2*^−/−^ double-mutant zebrafish also occurs with augmented seizure susceptibility that is corrected by rapamycin [116]. 

Likewise, *ubtor* is a vertebrate-specific, previously unannotated gene that encodes a protein whose depletion in cultured hippocampal neurons promotes neurite outgrowth and promotes mTOR signaling via its interaction with DEPTOR and mTOR complexes [117]. In mutant zebrafish, *ubtor* ablation increases spontaneous embryonic movement and neuronal activity in spinal interneurons, hyperactivation of mTOR signaling and sensitivity to pentylenetetrazole-induced seizures [117]. Importantly, such phenotypic abnormalities in zebrafish embryos and larvae are rescued by a conventional mTOR inhibitor rapamycin, collectively suggesting these fish as a valuable genetic model of mTORopathic epilepsy [117].

## 7. Discussion

While zebrafish hold much promise for studying mTORopathies and their genetic and molecular causes, these aquatic models also have certain significant limitations that must be considered. For example, the teleost-specific genome duplication may somewhat complicate genetic analyses of mTORopathies in zebrafish, since some mTOR-related genes (and those encoding their protein molecular interactors) may now exist in duplicates. 

Perhaps even more importantly, most clinical mTORopathies with epilepsy involve cortical malformations [118] (Table 1). On the one hand, this presents a general inherent conceptual limitation on the translatability of zebrafish data due to their lack of cortex and naturally lissencephalic brain [119]. On the other hand, some mTORopathies (e.g., LDD) involve cerebellar deficits and may be more easily recapitulated in zebrafish, given high homology between human and fish cerebellar structures. Another related problem is a key role of neurogenic stem cell polarity in [120], with a very distinct (by evagination) way of telencephalon formation in fishes [121]. Thus, it is possible that zebrafish may not be able to adequately reflect cortex-specific aspects of mTORopathic epilepsy. 

Furthermore, given such critical biological roles of the mTOR signaling pathway, it is also possible that mTORopathies are just too broad of an umbrella disorder group, which may not only be less specific (and, hence, diagnostically confusing) in terms of mimicking individual symptoms in animal models, but can also involve a substantial pathogenetic overlap with ‘downstream’ pathologies that may or may not directly stem from mTOR deficits per se (e.g., some channelopathies, tauopathies or gross neurodevelopmental disorders). As such, one subgroup of disorders may easily mask another related group when it comes to animal models, thus limiting their construct and predictive validity and complicating their dissection and translation into human conditions. 

Moreover, while a smaller size of brain in zebrafish (especially in larvae) vs. rodents presents a methodological challenge for assessing epilepsy, another limitation of using early neonatal larval fish is their not fully developed nervous, muscle and endocrine systems that may render them abnormally insensitive to selected epileptogenic drugs or molecular events. At the same time, most mTOR-related genes are highly homologous between fish, rodents and humans, reflecting the essential role of mTOR signaling for the cell in general. Thus, it is likely that mTORopathic epilepsy would still be possible to induce in fish genetically, and while its cortical aspects can be missed, the exact phenotypic manifestations of this pathology in zebrafish may differ from that of rodent or human mTORopathies. 

Notably, some of zebrafish pallial and subpallial areas correspond to various mammalian neocortical, hippocampal, striatal, pallidal and amygdalar structures. Given substantial genetic and neuroanatomical homology, zebrafish can therefore be broadly used and efficient in studies on evolutionarily conserved mechanisms of mTORopathies and their therapy. Indeed, as discussed above, the fact that zebrafish do display overt mTORopathic-like epileptic phenotypes seems to support this notion. 

## 8. Conclusions

In summary, mTORopathies represent an important group of severely debilitating neurological disorders whose commonly seen clinical aberrant phenotype includes pronounced, often treatment-resistant epilepsy (Figure 1 and Figure 2). Various animal models, only briefly discussed here, are a useful tool for studying molecular mechanisms of mTORopathic epilepsy. In addition to rodent models, mounting evidence suggests the zebrafish as a powerful novel organism for modeling mTORopathies. While there are still multiple open questions regarding conceptual and methodological approaches to studying epilepsy and other mTORopathies in zebrafish (Table 3), this aquatic species emerges as a promising tool for probing molecular mechanisms of mTOR-related epilepsy and for screening and developing potential novel antiepileptic therapies.

## Figures and Tables

**Table 1:** A brief summary of selected clinical mTORopathies (also see Figure 2).

Disorders, % Occurrence of Epilepsy	Affected Genes	Clinical Signs	Pathophysiology	Therapy of Epilepsy
Focal cortical dysplasia type IIB (FCDIIB), 5–25%	* MTOR *	Intractable seizures	Neuronal migration disruption by hyperactive mTOR, causing more cytomegalic neurons and spontaneous seizures	Antiepileptic drugs, surgical treatment
	* NPRL2, NPRL3, DEPDC5 *		Mutational inactivation, mTOR inhibition release and increased mTOR signaling	
Tuberous sclerosis complex (TSC), 80–90%	* TSC1 *	Subependymal nodules, giant cell tumors, cortical tubers, epilepsy	Overactivation of the mTOR kinase-dependent pathway	Antiepileptic drugs
	* TSC2 *	Benign tumors, epilepsy	Overactivation of the mTOR kinase-dependent pathway	
Hemimegaloencephaly (HME), >90%	*AKT1, AKT3, PIK3CA, PI3KR*	Enlarged heads of children, epilepsy, partial paralysis and cognitive delay	Mutation-facilitated mTOR signaling	Hemispherectomy
Glioneuronal tumors (GNT), >90%	*BRAF*	Early-onset focal epilepsy, chronic drug-resistant epilepsy	In V600E mutation, LKB1-induced phosphorylated LKB1 activates mTOR *	Surgical resection and radiation
Fragile X syndrome, 10–20%	*FMR1*	Intellectual disability, autism, seizures, hyper-activity, inattention, craniofacial deficits	FMR1/FMRP negative feedback loop causes mTOR inhibition	Symptomatic therapy (e.g., lithium, gabapentin)
Pretzel syndrome, >90%	*STRADA*	Severe developmental delay, epilepsy	A homozygous deletion of exons of the gene encoding the pseudokinase STRADA, an upstream inhibitor of mTOR	Sirolimus for the absent STRAD-alpha protein
Neuro-fibromatosis type 1, 5–10%	*NF1*	Neurocutaneous lesions, benign and malignant tumors, autism, occasional seizures	In primary cells lacking NF1 and in human malignancies, the mTOR pathway is severely downregulated	Surgical resection and radiation
Lhermitte-Duclos disease (LDD), >90% **	*PTEN*	Cerebellar gangliocytomas, ataxia, epilepsy	Due to increased PI3K/Akt activation and subsequent activation of the mTOR pathway, PTEN loss increases sensitivity to mTOR inhibitors	Surgical resection
TBCK syndrome, 70%	*TBCK*	Leukoencephalopathy, coarse face, neuropathy, hypotonia, intellectual disability, epilepsy	The mTOR complex’ protein levels significantly drop when TBCK is knocked down, and mTOR signaling is suppressed	Leucine (an mTORC1 activator)
Rett syndrome, 60–80%	*MECP2*	Speech deficits, uncontrollable movements, early-onset epilepsy	Mutation interrupts nucleolin–mTOR–P70S6K signaling	Antiepileptic drugs, deep brain stimulation
Beta-propeller protein-associated neurodegeneration (BPAN), 10%	*WDR45*	Intellectual disability, parkinsonism, psychomotor retardation, early-onset epilepsy, autism, dementia	The autophagy-inducing Unc51-like kinase 1 (ULK1)/Atg1-containing complex, controlled by the mTORC1	Ketogenic diet, vagal stimulation, behavioral therapy
Down syndrome, 1–15% ***		Intellectual disability, developmental delay, speech deficits and epilepsy	Hyperactivation of the AKT/mTOR signaling pathway, imbalances in autophagy and mitochondrial turnover	Antiepileptic drugs

* Likely by uncoupling the LKB1-AMPK-mTOR signaling. ** Difficult to estimate the exact occurrence due to a very rare nature of the disorder. *** Reaching 50% in those over 50.

**Table 2:** Summary of selected rodent models of mTORopathic epilepsy.

Disorder Modeled	Affected Genes	Details	Mechanisms/Pathway Affected	Cause of Epilepsy	References
Tuberous sclerosis (TSC)	*TSC2*	Tsc2+/− heterozygous mice (impaired memory and predisposition to seizures)	Overactivation of mTOR signaling due to TSC2 haploinsufficiency	Myelin delamination, larger brains with deficient connectivity	[83]
Pretzel syndrome	*STRADA*	Knockout mice, induced pluripotent stem cell-derived neurons from patients	Insufficient STRADA signaling via AMPK, leading to mTOR overactivation	Lower threshold for action potential, ectopic neurons in the subcortical white matter	[31]
Focal cortical dysplasia (FCD)	*PIK3CA H1047R*, *PIK3CA E545K*	Despite overt dysplasia, acute inhibition of PI3K signaling reduces epilepsy	Increased PI3K signaling, upregulation of pS473-Akt (mTOR-dependent)	Cortical malformation, hydrocephalic and enlarged brain	[43]
FCD type 2B (FCD2B) and TSC	*TSC2*	Oligodendroglial knockout in mice	Impeded oligodendrocyte development and myelin sheath formation	Impaired myelination	[39]
Fragile X syndrome (FXS)	*FMR1*	Knockout mice	Hippocampal mTOR phosphorylated and hyperactive	Affected synaptic plasticity, leading to overconnectivity	[85]

**Table 3:** Selected outstanding questions related to experimental modeling of mTORopathies.

Open Questions
Are there non-canonical, mTOR-independent mechanisms in epilepsy seen in mTORopathies? What is the exact role of neuronal vs. neuroglial (and also microglial vs. astrocytic) mechanisms in mTORopathies? Are there *disorder-specific* neurogenomic, neuroproteomic and neurometabolomic signatures of clinical mTORopathies? If yes, do zebrafish models for these specific mTOR-related disorders display similar ‘omics’ profiles to their human counterparts? Are there epigenetic mechanisms strongly involved in mTORopathic epilepsy? Can neuroinflammation modulate mTOR-related pathogenesis? What is the role of brain-derived neurotrophic factor (BDNF) and its signaling pathway in modulating mTORopathic epilepsy and other mTORopathies? What is the role of neuronal and neuroglial apoptosis in mTORopathic epilepsy? Can early-onset epilepsy (frequently seen in mTORopathies) trigger or promote neuroinflammation and neuronal apoptosis? Can these factors indirectly worsen mTORopathic pathogenesis progression further on? Can various psychological stressors (e.g., early-life, acute or chronic stress) alter the mTOR activity, to contribute to the development or progression of CNS deficits, including epilepsy? Can mTOR inhibitors represent novel therapies for neurodevelopmental disorders? Are there sex differences in mTOR activity in general, and in human, rodent and zebrafish epilepsy in particular? Can valid in vitro models of mTORopathic epilepsy be developed? How does diet (e.g., high-carbohydrate diet that predisposes to inflammation) impact the mTOR activity and, possibly, its role in epilepsy pathogenesis? Are there overt strain differences in mTOR activity in animal (e.g., mouse and/or zebrafish) models? Are there strain differences in mTORopathic epilepsy in such animal models? Which zebrafish strains are most susceptible to mTORopathic epilepsy? Do inbred (or, rather, outbred) zebrafish strains provide more reliable and reproducible phenotypic data related to mTORopathic epilepsy? What is the potential impact of ‘background’ fish strain on the expression of individual mTOR-related epileptic phenotypes in mutant zebrafish? To what extent can pharmacological manipulations used to induce epilepsy themselves affect mTOR signaling (e.g., pentylenetetrazole, a common chemoconvulsant, transiently activates mTOR activation in rats [122])? Inflammatory cytokines can induce aberrant mTOR activity (e.g., interleukins (IL) IL-1β, IL-17A and TNF-α strongly activate the mTOR kinase, PRAS40 and the downstream targets of mTOR activity 4E-BP1 and the ribosomal protein S6) [123]. Can drugs that upregulate such cytokines be used for the treatment of epilepsy (e.g., reducing glutamate reuptake, leading to elevated glutamate availability, and reducing inhibitory neurotransmission)? Epilepsy is a chronic disorder affecting all ages, but it peaks in the elderly [124]. Age itself can also impact mTOR activity [125]. Does age influence mTOR activity and, consequently, epilepsy in zebrafish and other animal models? How can zebrafish models contribute to probing the role of mutations at the mTOR inhibitor genes in neurodevelopmental disorders (e.g., autism, fragile X syndrome, tuberous sclerosis complex) that present epilepsy episodes as one of their common pathogenic symptoms? Can some novel therapies (e.g., resveratrol inhibiting mTOR through ATP competition [126]) be used as potential treatments for mTORopathic epilepsy? Can conventional antiepileptic drugs exert their auxiliary effects on mTOR signaling? Can there be a synergistic therapeutic action of the two potential mechanisms? Can mTOR-modulating drugs interact with antiepileptic drugs, either potentiating or mitigating their effects? Is there a complex link between other genetic mutations, mTOR activity, ionic channels activity and epilepsy? For example, mutations in the nitrogen permease regulator-like 2 gene (*NPRL2*) are linked to familial focal epilepsies, and its mutation affects the mTORC1 control of Na^+^ channel expression and brain amino acid homeostasis, which both can contribute to epilepsy pathogenesis [127]. Are there potential peripheral biochemical biomarkers of mTORopathic epilepsy in zebrafish and other animal models?

## Data Availability

Not applicable.
